# A 2-year-old girl with co-inherited cystic fibrosis and sickle cell-β^+^ thalassemia presenting with recurrent vaso-occlusive events during cystic fibrosis pulmonary exacerbations: a case report

**DOI:** 10.1186/1752-1947-7-203

**Published:** 2013-07-26

**Authors:** Kurtis T Sobush, Courtney D Thornburg, Judith A Voynow, Stephanie D Davis, Stacey L Peterson-Carmichael

**Affiliations:** 1 Division of Pediatric Pulmonary Medicine, Saint Louis University School of Medicine, Saint Louis, Missouri, USA; 2 Division of Pediatric Hematology/Oncology, Duke University Medical Center, Durham, North Carolina, USA; 3 Division of Pediatric Pulmonary and Sleep Medicine, Duke University Medical Center, Durham, North Carolina, USA; 4 Division of Pediatric Pulmonary-Critical Care, Riley Hospital for Children at Indiana University Health, Indianapolis, Indiana, USA; 5 Division of Pediatric Critical Care, Duke University Medical Center, Box 3046, Erwin Road, Durham, North Carolina 27710, USA

**Keywords:** Acute chest syndrome, Cystic fibrosis, Hemoglobinopathy, Infant pulmonary function testing, Raised volume rapid thoracoabdominal compression, Sickle cell, Thalassemia, Vaso-occlusive event

## Abstract

**Introduction:**

This is the first published report of a young girl with co-inherited sickle cell-β^+^ thalassemia and cystic fibrosis. Although a small subset of patients with co-inherited cystic fibrosis and other hemoglobinopathies have been reported, this patient developed early hematologic and pulmonary complications that were more severe than the previous cases. To assess pulmonary co-morbidities, we used infant pulmonary function testing through the raised volume rapid thoracoabdominal compression technique as both an established study of early cystic fibrosis and also as a newer study of mechanism for early sickle cell lung disease. This further serves as the first report of the raised volume rapid thoracoabdominal compression technique to determine raised volume forced expiratory flows and fractional lung volumes in a patient with a hemoglobinopathy.

**Case presentation:**

A 2-year-old African-American girl with co-inherited cystic fibrosis and sickle cell-β^+^ thalassemia developed severe hematologic complications (recurrent vaso-occlusive events, hepatic sequestration, and acute chest syndrome) during periods of cystic fibrosis pulmonary exacerbations and weight loss. Because cystic fibrosis and sickle cell-β^+^ thalassemia both confer distinct patterns of pulmonary disease, infant pulmonary function testing with the raised volume rapid thoracoabdominal compression technique was used to define respiratory pathophysiology and guide treatment options. Infant pulmonary function testing data demonstrated moderate-to-severe lower airways obstruction, moderate air trapping, and no evidence of restrictive lung disease.

**Conclusions:**

Infant pulmonary function testing with the raised volume rapid thoracoabdominal compression technique guided therapy in this patient with cystic fibrosis and sickle cell-β^+^ thalassemia. Although this is an original case report on a unique patient, this case highlights the need to evaluate early respiratory pathophysiology in a broader population of young patients with hemoglobinopathies and screen those at risk for early pulmonary co-morbidities.

## Introduction

Cystic fibrosis (CF) and sickle cell-β^+^ thalassemia (S-β^thal^) are both autosomal recessive conditions with associated acute and chronic pulmonary morbidities such as acute chest syndrome (ACS), sickle chronic lung disease (SCLD), and CF-related bronchiectasis. Multiple early interventions have been instituted in an attempt to reduce the incidence and severity of respiratory complications associated with SCLD and CF that often start in infancy. These have included newborn screening programs for CF and hemoglobinopathies along with referral to subspecialty physicians and established care programs focused on minimizing anticipated complications. In recent years, infant pulmonary function testing (IPFT) using the raised volume rapid thoracoabdominal compression (RVRTC) technique has been applied as a clinical tool and research aid to assess early airway obstruction and air trapping in infants with CF [1] and in infants with cough or wheeze unresponsive to bronchodilators [2]. There are no published data on infants with sickle cell disease (SCD) using the RVRTC technique and there is only one study detailing lung function measures in infants with SCD using a partial raised volume technique with findings suggestive of lower airways obstruction [3]. The advantage of the RVRTC technique over either partial expiratory flow or tidal breathing maneuvers is that flows are assessed over much larger lung volumes and there is achievement of flow limitation. Thus, the measures are more likely to be effort-independent; thereby reflecting underlying lung mechanics [4].

We present a novel case of a young girl with co-inherited CF and S-β^thal^. This patient developed multiple CF pulmonary exacerbations (defined for this patient by fever presence, increased cough, new infiltrate on chest radiograph, and initiation of intravenous antibiotics) and ACS (defined by the presence of rapid onset of fever, hypoxia, new infiltrate on chest radiograph, and any respiratory symptoms). IPFT measures using the RVRTC technique were obtained to help define her respiratory pathophysiology. This is the first known report of raised volume forced expiratory flow measures and fractional lung volumes in a young child with CF and a hemoglobinopathy. We will highlight considerations for management in this rare population and how this was guided, in part, by infant lung function testing.

## Case presentation

A 17-day-old African-American girl was admitted for management of failure to thrive and general malaise. The initial workup was guided, in part, by an abnormal newborn hemoglobin electrophoresis screen consistent with SCD. Repeat electrophoresis confirmed a diagnosis of S-β^thal^. In addition to the patient’s hematologic workup, the patient was found to have a severely low fecal elastase level (<50μg elastase/g stool) consistent with pancreatic insufficiency. Subsequent sweat testing was performed and was positive on two occasions (73 and 72mmol/L with first test; 88 and 103mmol/L with second test) confirming a diagnosis of CF. CF genotyping results were positive for one intronic mutation for C.53+2 T>C; a second mutation was not identified.

This patient, who is now 4 years of age, has required 15 hospitalizations (most during the first 2 years of life) to manage complications of both CF and S-β^thal^. In general, the majority of hospitalizations were for presumed CF pulmonary exacerbations or weight loss. At 2 years of age, the patient began to develop vaso-occlusive events (VOE) including multiple bouts of pain crises and hepatic sequestration contributing to prolonged hospital stays for CF pulmonary exacerbations. Pain crises involving the chest, back, and abdomen limited both airway clearance and nutritional support with enteral feedings. Furthermore, the patient faced acute respiratory decline during VOE with hypoxia, fever, wheezing, and a new pulmonary infiltrate on chest radiograph consistent with ACS. The underlying etiology of ACS development was unclear although it was attributed to complications of CF, S-β^thal^, or both. Along with computed tomography imaging of the chest (mild bronchiectasis with bibasilar opacities consistent with atelectasis) and flexible bronchoscopy (normal airway anatomy with diffuse, viscous white secretions with 2,704 cells/mm^3^ and 51% neutrophils), IPFT measures were obtained to better define respiratory physiology and guide a treatment plan.

**Figure 1:**
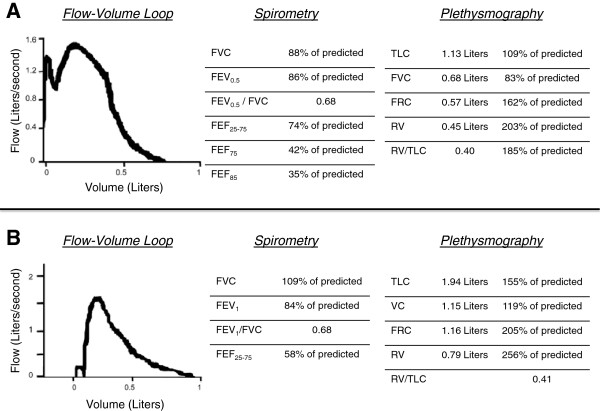
**Pulmonary function testing data. A**. Acquired at 2 years of age using the raised volume rapid thoracoabdominal compression technique along with whole body plethysmography. Normative data by Castile *et al*. and Jones *et al*. [4,6]. **B**. Acquired at 4 years of age using classical spirometry and whole body plethysmography. FVC- Forced vital capacity; FEV_0.5_ - Forced expiratory volume in 0.5 second; FEF_25-75_ - Forced expiratory flow between 25 and 75% of the FVC; FEF_75_ - Forced expiratory flow at 75% of the FVC; FEF_85_ - Forced expiratory flow at 85% of the FVC; TLC- Total lung capacity; FRC- Functional residual capacity; RV- Residual volume; FEV_1_ – Forced expiratory volume in 1 second; VC- Vital capacity.

Infant lung function testing was performed using the RVRTC technique which elevates the lung volume of infants to near total lung capacity prior to performing a rapid thoracic compression maneuver. These forced expiratory flows from raised lung volumes were obtained after sedation with chloral hydrate according to American Thoracic Society/European Respiratory Society guidelines and approximate adult-type lung function maneuvers [5]. Post-bronchodilator results were not obtained due to lack of sedative effects near the end of testing. IPFT data (Figure 1A) demonstrate moderate-to-severe lower airways obstruction, moderate air trapping, and no evidence of restrictive lung disease.

The IPFT data prompted initiation of inhaled deoxyribonuclease (DNase) in an effort to reduce any distal neutrophilic mucus impaction contributing to air trapping. In addition, due to the combination of frequent VOE, ACS, and new evidence of lower airways obstruction, hydroxyurea was started to attempt to theoretically minimize progression of SCLD [7]. Although hydroxyurea is predominantly utilized in patients with more severe forms of SCD, clinical response has been shown in patients with S-β^thal^[8]. Chronic transfusion therapy was considered but is not optimal given the risks of iron overload, autoantibody and alloantibody formation, and human leukocyte antigen sensitization that could complicate future stem cell and/or lung transplantation. Hospitalization frequency has subsequently improved with a reduction from 3.5 admissions/year to 0.5 admission/year following IPFT and therapeutic interventions. Spirometry data obtained at 4 years of age is included for comparison in Figure 1B. Although expiratory time is short, flow-volume loops reflect evidence of effort-independent flow limitation and suggest continued moderate lower airways obstruction with significant response to bronchodilator. Inhaled budesonide was initiated following spirometry secondary to the findings of bronchodilator responsiveness.

## Discussion

To the best of our knowledge, this is the first reported case of a patient with co-inherited CF and S-β^thal^. Only four previous case reports have described a subset of five patients with co-inherited CF and a hemoglobinopathy (Table 1) [9-12]. In the previous cases, lung function remained relatively well preserved. Furthermore, pulmonary and hematologic complications were not concurrent. By contrast, the patient in this case report showed significant loss of lung function at an early age as demonstrated by IPFT. In addition, CF pulmonary exacerbations frequently occurred in temporal association with ACS and pain crises.

**Table 1:** Current and prior case reports of co-inherited cystic fibrosis and sickle cell disease

**Reference**	**Cystic fibrosis genotype**	**Cystic fibrosis complications**	**Hemoglobin disease classification**	**Hematologic complications**	**Co-morbid conditions**
Porter *et al*. [9]	Unknown	-Meconium ileus	Hemoglobin SS	-Acute transfusions	-None reported
Porter *et al*. [9]	Unknown	-Recurrent bronchitis	Hemoglobin SS	-Anemia	-None reported
Amendola *et al*. [10]	Unknown	-Recurrent bronchitis	Hemoglobin SD	-Chronic transfusions	-None reported
		-Pancreatic insufficiency			
Warwick *et al*. [11]	Unknown	-Recurrent bronchitis	Hemoglobin SS	-Bone pain	-Cardiomegaly
		-Pancreatic insufficiency			
Banjar [12]	H_549_L/S_549_R	-Sinusitis	Hemoglobin SS	-Bone pain	-Obstructive sleep apnea
		-Recurrent bronchitis		-Chronic transfusions	-Hypercalciuria
		-Pancreatic insufficiency			-Gastroesophageal reflux
Current case	C_53+2_T>C/Unknown	-Recurrent bronchitis	Hemoglobin-β^+^ thalassemia	-Bone pain	-Gastroesophageal reflux
		-Failure to thrive		-Abdominal sequestration	-Nissen fundoplication
		-*Burkholderia cepacia*		-Acute chest syndrome	-Gastrostomy tube
		-Pancreatic insufficiency		-Chronic transfusions	-Central line infections
					-Lactase deficiency
					-Total parenteral nutrition

The rationale for concurrent pulmonary and hematologic complications is probably multifactorial and may be explained due to a combination of pathophysiologic effects. Intraluminal airway secretions are known to have different cellularity in CF bacteriologic bronchitis compared to plastic bronchitis associated with ACS. If airway secretions are available from sputum collection or bronchoalveolar lavage, formal pathologic assessment may guide therapeutic options based upon cellular and/or fibrinous appearance. In addition, *in vitro* assessment of airway secretions exposed to different therapeutic modalities may help guide therapy. DNase has been shown in both CF and ACS to aid in airway clearance [13-15]. Additional small case reports have evaluated the potential utility of inhaled N-acetylcysteine [14,16], along with inhaled tissue plasminogen activator [17] although data is sparse with these therapeutic options. The patient in this report was unable to provide expectorated sputum samples due to her age. Bronchoalveolar lavage fluid was not obtained during acute pulmonary exacerbations secondary to concerns for worsening atelectasis and escalating oxygen needs after general anesthesia. Regardless of the cellular makeup of the intraluminal secretions, it is probable that either would cause regional changes in oxygenation, both of which could precipitate the development of ACS.

Another complicating factor was the need for high dose opioid narcotics during pain crises. Opioid narcotics may further contribute to generalized histamine release that may contribute to airway hyperresponsiveness or increased airway edema. Use of aggressive narcotics for pain to allow airway clearance was favored over the possibility of worsened lower airways obstruction due to a direct opioid effect. Analgesic alternatives including intravenous ketorolac and transdermal lidocaine were utilized in the interest of minimizing opioid dosing.

The approach to managing an acute respiratory decline proved difficult in this patient due to the multiple disease processes involved. IPFT was utilized to help delineate various possible pathophysiologic mechanisms: lower airways obstruction, regional air trapping, and/or restrictive lung disease. Children with SCD are more likely to have wheezing and symptoms of airway hyperresponsiveness during episodes of ACS and have a higher incidence of asthma than the general population. Data supports an association between asthma and increased frequency of ACS in older patients with SCD however the temporal relationship has not been defined [18]. As these patients age, they often develop SCLD, a restrictive lung disorder as evidenced by adult pulmonary function data [19]. With the multiple episodes of ACS in this case, there was an unexpected lack of restrictive lung disease according to the IPFT data. Data are lacking regarding the incidence and severity of lung disease in young children with SCD. A multicenter clinical trial addressing this question would be of immense value to the sickle cell population and the clinical teams caring for these children.

## Conclusions

Although the co-inheritance of CF and a hemoglobinopathy is relatively rare, the use of infant lung function testing as detailed in this report may be of benefit to a broader population of young children with SCD. With the advanced technology allowed by the RVRTC technique, young patients with SCD may have their lung function followed longitudinally. Should this population have evidence of early lung disease, IPFT could become an additional screening tool to allow for earlier interventions that could minimize the progression of chronic lung disease in SCD.

## Consent

Since the patient described in this report is a minor, the authors have obtained written informed consent from the patient’s legal guardian for publication of this case report and accompanying images. A copy of this written consent is available for review by the Editor-in-Chief of this journal.

## Abbreviations

ACS: Acute chest syndrome; CF: Cystic fibrosis; IPFT: Infant pulmonary function testing; RVRTC: Raised volume rapid thoracoabdominal compression; S-βthal: Sickle cell-β^+^ thalassemia; SCD: Sickle cell disease; SCLD: Sickle chronic lung disease; VOE: Vaso-occlusive events.

## Competing interests

The authors declare that they have no competing interests.

## Authors’ contributions

KS collected and analyzed infant pulmonary function case data and was the major contributor in writing the manuscript. CT provided critical manuscript revision and insight pertaining to the hematologic manifestations of this case. JV was involved in intellectual critique of the clinical pulmonary aspects of this case. SD provided critical manuscript revision pertaining to IPFT performed on this patient. SPC collected and analyzed infant pulmonary function case data, contributed to certain aspects of the written manuscript, and offered critical revision pertaining to pulmonary aspects of this case, in particular as it related to IPFT. All authors read and approved the final manuscript.
